# Identification of Stemness Characteristics Associated With the Immune Microenvironment and Prognosis in Gastric Cancer

**DOI:** 10.3389/fonc.2021.626961

**Published:** 2021-03-03

**Authors:** Deli Mao, Zhijun Zhou, Shenglei Song, Dongsheng Li, Yulong He, Zhewei Wei, Changhua Zhang

**Affiliations:** ^1^Digestive Diseases Center, The Seventh Affiliated Hospital of Sun Yat-sen University, Shenzhen, China; ^2^Department of Medicine, The University of Oklahoma Health Sciences Center, Oklahoma City, OK, United States; ^3^Department of Gastrointestinal Surgery, The First Affiliated Hospital of Sun Yat-sen University, Guangzhou, China

**Keywords:** gastric cancer, stemness index, prognostic signature, tumor microenvironment, immune response

## Abstract

**Background:**

Gastric cancer (GC) is a highly heterogeneous disease. In recent years, the prognostic value of the mRNA expression-based stemness index (mRNAsi) across cancers has been reported. We intended to identify stemness index-associated genes (SI-genes) for clinical characteristic, gene mutation status, immune response, and tumor microenvironment evaluation as well as risk stratification and survival prediction.

**Methods:**

The correlations between the mRNAsi and GC prognosis, clinical characteristics, gene mutation status, immune cell infiltration and tumor microenvironment were evaluated. Weighted gene correlation network analysis (WGCNA) was performed to identify SI-genes from differentially expressed genes (DEGs) in The Cancer Genome Atlas (TCGA). Single-sample gene set enrichment analysis (ssGSEA) was employed to calculate the sample SI-gene-based ssGSEA score according to the SI-genes. Then, the correlations between the ssGSEA score and GC prognosis, clinical characteristics, gene mutation status, immune cell infiltration and tumor microenvironment were analyzed. Finally, the least absolute shrinkage and selection operator (LASSO) Cox regression algorithm was used to construct a prognostic signature with prognostic SI-genes. The ssGSEA score and prognostic signature were validated using the Gene Expression Omnibus (GEO) database.

**Results:**

The mRNAsi could predict overall survival (OS), clinical characteristics, the gene mutation status, immune cell infiltration, and the tumor microenvironment composition. Fourteen positive SI-genes and 178 negative SI-genes were screened out using WGCNA. The ssGSEA score, similar to the mRNAsi, was found to be closely related to OS, clinical characteristics, the gene mutation status, immune cell infiltration, and the tumor microenvironment composition. Finally, a prognostic signature based on 18 prognostic SI-genes was verified to more accurately predict GC 1-year, 3-year, and 5-year OS than traditional clinical prediction models.

**Conclusion:**

The ssGSEA score and prognostic signature based on 18 prognostic SI-genes are of great value for immune response evaluation, risk stratification and survival prediction in GC and suggest that stemness features are crucial drivers of GC progression.

## Introduction

Gastric cancer (GC) was ranked fifth in incidence among all cancers and third in cancer-related deaths in 2018. With the eradication of *Helicobacter pylori*, the incidence and mortality of GC have decreased in recent years (1, 2). However, according to an epidemiological survey conducted in 2018, there are still 1,033,701 new cases of GC and 782,685 GC-related deaths every year worldwide (3). The high incidence and mortality of GC place a substantial burden on the social economy, especially in Asian countries, such as South Korea, Japan, and China. The majority of patients are already at an advanced stage at the time of diagnosis, such as those in China, which causes the mortality rate of GC in most parts of the world to remain above 75% (4). Therefore, accurate prognostic evaluation, postoperative follow-up, and timely intervention are of utmost importance. At present, histopathological classification is commonly used clinically to predict the outcomes of GC patients. However, studies have shown that the impact of grade on prognosis varies greatly depending on the tumor site and patient age (5). The same types of tumors also have great variability due to their cytological features and architecture, resulting in different histological classifications (6). In addition, the International Union Against Cancer classification, Ming classification, Borrmann classification, and Laurén classification have all been proposed (7). These histopathological classification systems predict the prognosis of GC at the level of pathological features, but it is often difficult to accurately predict patient outcomes. We know that the occurrence and development of GC are the result of the accumulation of multiple molecular changes. Only a thorough understanding of the mechanism of cancer can better predict terminal events and guide clinical treatment. Therefore, the development of novel molecular biomarkers for GC genetic classification is urgently needed.

Tumor growth is maintained by extremely limited self-renewing stem cells. These cancer stem cells (CSCs) are generally in a dormant state but are easily activated after radiotherapy or chemotherapy to promote tumor invasion and metastasis (8). The activation of CSCs is also an important cause of chemotherapy resistance (9). Vermeulen et al. used a cancer stem cell model in 2012 to explain the mechanism of tumor metastasis and drug resistance in detail (10). GC stem cells were first isolated in 2007 to study the interaction mechanism between *Helicobacter pylori* and tumor cells, and a *Helicobacter pylori* culture that upregulated the expression of telomerase in GC stem cells was discovered (11). In the past 10 years, research on GC stem cells has discovered many possible signaling pathways (12) and potential stem cell biomarkers (13, 14), prompting us to conclude that stem cells have a profound impact on the prognosis of GC patients. In recent years, stemness indices have been calculated to indirectly describe stemness features. The degree of oncogenic dedifferentiation was evaluated by Malta et al. (15) using a machine learning algorithm to calculate stemness indices for pluripotent stem cell samples. Studies have shown that the mRNA expression-based stemness index (mRNAsi) is closely related to the prognosis of GC, which provides new insights for predicting GC tumor outcomes, recurrence, and metastasis. The slight shortcoming of that study was that it aimed to evaluate the correlation between the mRNAsi and pan-cancer data. Pluripotent stem cell samples were used to evaluate the mRNAsi values of GC; however, this complicated method is not suitable for clinical application. Hence, based on this research, we used bioinformatic algorithms to focus on the prognostic value of the GC mRNAsi and stemness index-associated genes (SI-genes) that affect mRNAsi values, and single-sample gene set enrichment analysis (ssGSEA) and a logistic regression risk prediction model were employed to explore a novel stemness index-associated signature to accurately predict the prognosis and tumor stratification of GC.

## Materials and Methods

### Data Sources and Processing

The RNA-seq profile of 375 patients with GC and their clinical information were downloaded from The Cancer Genome Atlas (TCGA) website (https://portal.gdc.cancer.gov/). The GTF annotation file was downloaded from the Ensembl Genome Browser (http://asia.ensembl.org/index.html) to convert the Ensembl gene ID into the gene symbol and extract the mRNA profile. Two microarray cohorts, GSE66229 and GSE15459, were enrolled in our study. The expression profile and clinical information of the microarray cohorts were acquired from the GEO database (https://www.ncbi.nlm.nih.gov/geo/). Then, the ComBat method was used to remove batch effects by the R package “sva”. The TCGA-STAD somatic mutation data were obtained from the TCGA website. The mutation status was identified and visualized using the R package “maftools”. The tumor mutational burden (TMB) was defined as the total number of mutations per megabase in tumor tissue, including base substitutions, deletions, insertions, and coding errors, which were extracted and estimated by Perl scripts (**Supplementary Table 1**). In addition, PD-L1 protein expression data for GC (level 3) was obtained from The Cancer Proteome Atlas (TCAP) (https://tcpaportal.org/tcpa/index.html). Finally, the mRNAsi of GC was provided by Malta et al. (15) using a one-class logistic regression machine learning algorithm, which was obtained from the NIH Genomic Data Commons (https://gdc.cancer.gov/about-data/publications/PanCanStemness-2018). The TCGA cohort was set as the training group for this study, and the GSE66229 and GSE15459 datasets were set as the validation cohorts.

### Immune Cell Infiltration and the Tumor Microenvironment Score

Tumor IMmune Estimation Resource (TIMER) (http://timer.cistrome.org/) is a web server for comprehensive analysis of tumor-infiltrating immune cells. TIMER (16), CIBERSORT (17), and EPIC (18) methods were employed to evaluate infiltrating macrophage, M2 macrophage, and cancer-associated fibroblast (CAF) abundances based on a GC mRNA expression profile. The tumor microenvironment score of each sample, including the stromal score, immune score, ESTIMATE score, and tumor purity, was calculated by the package “estimate” in R according to the GC mRNA expression profile.

### Weighted Gene Correlation Network Analysis

WGCNA aims to identify coexpressed gene modules and explore the relationships between gene networks and a phenotype of interest, as well as investigate the core genes in a network. Before performing WGCNA, we used the “limma” R package to screen differentially expressed genes (DEGs) in GC between tumor tissue and normal tissue in the TCGA cohort. The filter conditions were | logFC |>1 and adj. PValue<0.05. The WGCNA was conducted with the “WGCNA” package. First, the correlation coefficient between any two genes was calculated, and the weighted value of the correlation coefficient was used to make the connections among the genes in the network obey scale-free networks. Then, a hierarchical clustering tree was constructed from the correlation coefficients between genes. Different branches of the clustering tree represent different gene modules, and different colors represent different modules. Next, module significance (MS) was calculated and used to measure the correlations of an mRNAsi value with the different modules and record the genes in each module. The genes in each module were considered module eigengenes (MEs). The correlations between an mRNAsi value and genes were measured by gene significance (GS). Module membership (MM) was defined as the correlation between a DEG expression profile and the module genes. In addition to the mRNAsi, the epigenetically regulated mRNAsi (EREG-mRNAsi) was also selected as the clinical phenotype. The module with the minimum MS value was regarded as the negative module, and the module with the maximum MS value was regarded as the positive module. After selecting the module of interest based on the MS value, SI-genes were screened according to the previously reported standard (19): GS value>0.5 and MM value> 0.8. The SI-genes in the negative module were used as negative SI-genes, and the SI-genes in the positive module were used as positive SI-genes. Module-trait relationships were estimated using Pearson’s correlation analysis between the module eigengene and the values of the mRNAsi and EREG-mRNAsi, which allowed easy identification of the mRNAsi values highly correlated with the expression set.

### Gene Set Enrichment Analysis

The possible signaling pathways involved in GC progression were explored using GSEA performed with GSEA software. mRNAsi values were used as the phenotype, and “hallmark gene sets” were downloaded from the Molecular Signatures Database (MSigDB, v7.2) (http://software.broadinstitute.org/gsea/msigdb/). Pathways were considered statistically significant with an FDR < 0.25.

Positive and negative SI-genes were previously identified through WGCNA. Then, ssGSEA was applied to calculate a sample SI-gene-based ssGSEA score with the R package “GSVA”, and the SI-gene-based ssGSEA score of each sample was equal to the positive SI-gene-based ssGSEA score minus the negative SI-gene-based ssGSEA score.

### Construction of a Prognostic Signature

Genes that were highly correlated with prognosis and crucial were identified by univariate Cox regression analysis, and a forest plot was drawn using the “survival” package. The least absolute shrinkage and selection operator (LASSO) Cox regression algorithm was used to select prognostic SI-genes and calculate variable coefficients with the “glmnet” package. Then, we calculated the riskScore of each sample according to the following formula:

$$\text{riskScore}=\overset{n}{\underset{i=1}{\sum }}Coef_{i}\times X_{i}$$

where *coef* is equal to the gene coefficient, and *X* represents the gene expression level. The median value of the riskScore for all samples in the TCGA cohort was taken as the cut-off value. According to the cut-off value, the samples in the training and validation cohorts were divided into high- and low-risk groups.

### Prognostic Value of the Prognostic Signature

Kaplan-Meier survival analysis was applied to compare the overall survival (OS) of GC patients in the high- and low-risk groups. Receiver operating characteristic (ROC) curve analysis was performed to detect the sensitivity and specificity of the riskScore in predicting OS. Univariate and multivariate Cox regression analyses were applied to evaluate whether clinical characteristics and the riskScore are risk factors for the prognosis of GC and to calculate the hazard ratio (HR) with the R package “survival”. A nomogram was plotted to predict the of 1-, 3-, and 5-year OS GC patients by the R package “regplot”. A calibration curve was drawn to compare the difference between the predicted survival probability and observed survival probability using the “rms” and “foreign” packages. The concordance index (C-index) calculated with the “rms” package was used to reflect the ratio of the predicted result to the actual result. Decision curve analysis (DCA) (20) was performed to describe the potential clinical impact of the prognostic signature and compare it with the benefit rate of a single indicator. On this basis, a clinical impact curve (CIC) was drawn by the R package “rmda” to predict risk stratification. The net reclassification index (NRI) (21) was calculated with the “nricens” package to compare the predictive capabilities of the new model and old models. Integrated discrimination improvement (IDI) was evaluated to examine the overall improvement represented by the new model compared to an old model using the R package “PredictABEL”.

### Statistical Analysis

The Wilcoxon test was used to compare differences between two groups of nonnormally distributed data. A two-tailed unpaired Student’s t test was used to compare differences between two groups of normally distributed data. Differences between rates were tested by the chi-square test or Fisher’s exact test (n>40 for the chi-square test, and n ≤ 40 for Fisher’s exact test). Kaplan-Meier curve analysis was performed to compare differences in prognosis between two groups of patients. Pearson’s correlation analysis was performed to compare correlations between two sets of data and calculate the correlation coefficient. R software (version 3.6.3), SPSS 22.0, and Prism 8 were used for statistical analysis and graphing. *P*<0.05 was considered statistically significant.

## Results

### Data Processing

**Figure 1:**
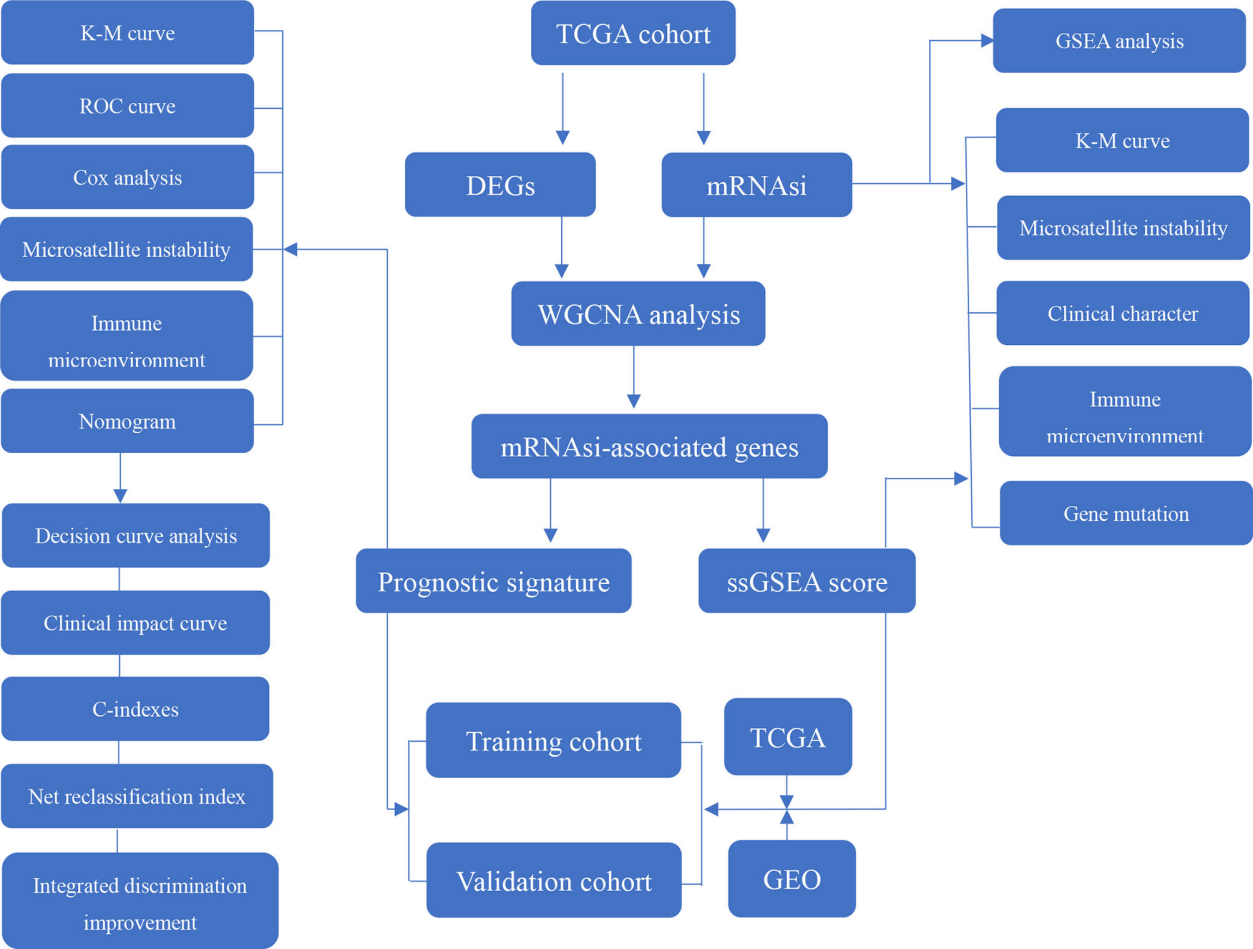
Flow diagram presenting the main plan and process of the study.

This study procedure was conducted methodically based on the steps outlined in the flow diagram (**Figure 1**). To make the results of this study sufficiently reliable, a TCGA dataset was categorized as a training cohort, GSE66229 (n = 300) and GSE15459 (n = 200) were categorized as validation cohorts, and the corresponding clinicopathological characteristics were extracted (**Table 1**). The training cohort included 32 normal tissue samples and 375 tumor tissue samples. Transcriptome profiling was standardized using the “limma” package. Next, we used Perl software to select an mRNA expression microarray from the transcriptome profiling. At the same time, the mRNAsi corresponding to the tumor tissue samples was extracted from pan-cancer mRNAsi datasets (**Supplementary Table 2**).

**Table 1:** Clinicopathological characteristics of GC patients from the TCGA and GEO cohorts.

Characteristics	TCGA training cohort(n = 375)	GSE66229 validation cohort(n = 300)	GSE15459 validation cohort(n = 200)
Sex			
Female	134	101	67
Male	241	199	124
Unknown	0	0	9
Survival status			
Alive	201	148	96
Dead	174	152	95
Unknown	0	0	9
Age (years)			
≤60	121	117	60
>60	250	183	131
Unknown	4	0	9
T stage			
T1	19	0	NA
T2	80	188	
T3	168	91	
T4	100	21	
Unknown	7	0	
N stage			
N0	114	38	NA
N1	99	131	
N2	76	80	
N3	74	21	
Unknown	12	0	
M stage			
M0	347	273	NA
M1	25	27	
Unknown	3	0	
Stage			
Stage I	54	30	31
Stage II	116	97	29
Stage III	151	96	72
Stage IV	39	77	59
Unknown	15	0	9

### Predicting Outcomes and Clinical Characteristics Using the mRNAsi

**Figure 2:**
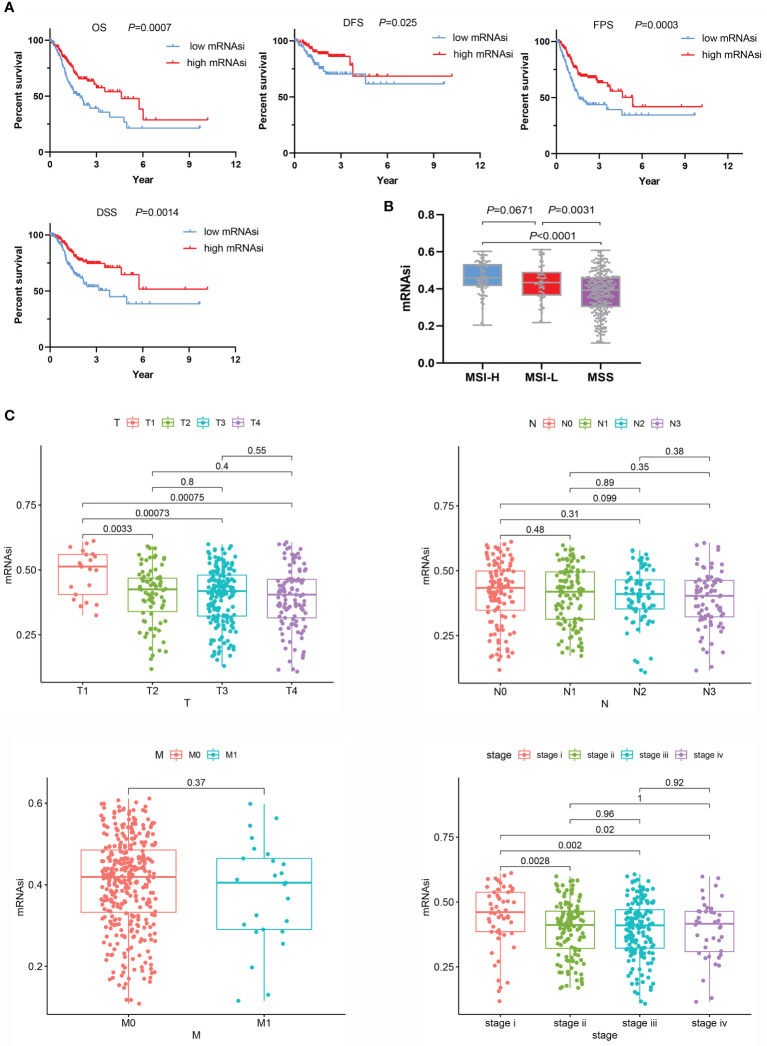
Relationships between the mRNAsi and clinicopathological characteristics. **(A)** In the group with high mRNAsi values, the OS, DFS, PFS, and DSS of GC patients were better than those in the low mRNAsi value group. **(B)** Evaluation of mRNAsi values on the basis of MSS. **(C)** mRNAsi values were associated with clinical characteristics.

First, a Kaplan-Meier curve was plotted to observe the effect of mRNAsi values on the prognosis of GC patients. Patients with higher mRNAsi values had prolonged OS (*P*=0.007) and disease-free survival (DFS) (*P*=0.025) (**Figure 2A**). These results were consistent with the results of Malta et al. (15) (OS: *P*<0.05, HR<1; DFS: *P*<0.05, HR<1). In addition, this study further found that mRNAsi values affected progression-free survival (PFS: *P*=0.0003, HR<1) and disease-specific survival (DSS) (*P*=0.0014, HR<1) (**Figure 2A**). Microsatellite instability (MSI) is an important indicator that affects the response to chemotherapy and prognosis of GC. A large-sample multi-center meta-analysis reported that patients with MSI are more likely to benefit from treatment than those with microsatellite stability (MSS) (22). There was also a close relationship between mRNAsi values and MSI, and mRNAsi values were higher in the MSI-H and MSI-L groups than in the MSS group (*P*<0.001) (**Figure 2B**). Groups with high mRNAsi values had a higher incidence of MSI and were able to achieve better chemotherapy responses and thus exhibited a better prognosis. This explanation was consistent with the previous meta-analysis. Then, we compared differences between mRNAsi values and clinicopathological characteristics. The mRNAsi values for the pathologic T2, T3, and T4 stages were significantly lower than those for the pathologic T1 stage (*P*<0.01). The same phenomenon was observed for the pathologic tumor stage. The mRNAsi values for the pathologic tumor stage II, III, and IV groups were significantly lower than those for the stage I group (*P*<0.05). However, the mRNAsi value distinction was not seen for different pathologic N or M stages (**Figure 2C**).

### mRNAsi Evaluated in the Context of the Tumor Microenvironment

**Figure 3:**
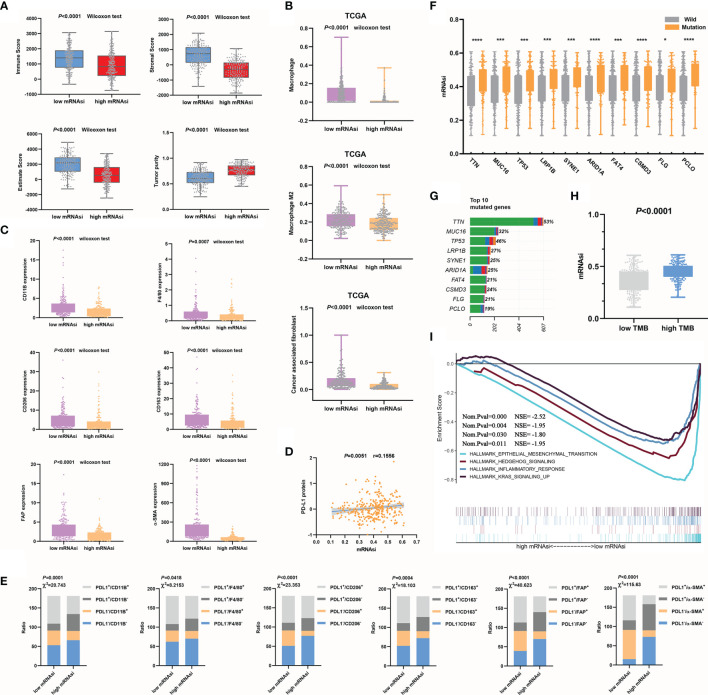
Relationships between mRNAsi values and the mutation status or tumor microenvironment. **(A)** Higher mRNAsi values corresponded to a lower immune score, stromal score, and ESTIMATE score and higher tumor purity. **(B, C)** mRNAsi values were closely related to macrophages, CAFs **(B)** and their markers **(C)**. **(D)** mRNAsi values were used to evaluate the efficacy of immunotherapy in GC. **(E)** The correlations between mRNAsi values and immune cell types **(F–G)** The top 10 mutant genes in GC **(G)** and their correlations with the mRNAsi are shown, *P<0.05; ***P<0.001; ****P<0.0001 **(F)**. **(H)** The mRNAsi values in the high TMB group were significantly increased. **(I)** EMT signaling was significantly enriched during GC progression.

We found a strong association between the mRNAsi and a known tumor microenvironment composition. Tumor tissues with a relatively high mRNAsi often contained fewer immune and stromal components and the ESTIMATE score. However, in the high mRNAsi group, tumor purity was higher (**Figure 3A**). We also computed the correlations of tumor microenvironment compositions with the mRNAsi by Pearson’s correlation analysis. mRNAsi values showed obvious negative correlations with immune scores (*P*<0.0001, r=-0.3421), stromal scores (*P*<0.0001, r=-0.7561) and ESTIMATE scores (*P*<0.0001, r=-0.5980). For the mRNAsi, higher positive correlation values were seen with tumor purity (*P*<0.0001, r=0.5976) (**Supplementary Figure 1A**).

We further clarified the correlations between the mRNAsi and immune microenvironment variables in the context of the immune cell subtypes of tumors. Significantly increased macrophage and CAF infiltration was observed in GC samples with decreased mRNAsi values compared with those with increased mRNAsi values (**Figure 3B**). Additionally, to make the analysis more reliable, we tested the expression of the macrophage markers CD11B, F4/80 (23), CD206, and CD163. Among these markers, CD206 and CD163 are mainly M2 macrophage markers (24). The CAF markers FAP and α-SMA (25) were also examined. The results showed that the expression of these characteristic markers (**Figure 3C**) was also decreased in the high mRNAsi value group compared to the low mRNAsi value group. Anti-PD-L1 and anti-PD-1 antibodies are currently considered to be relatively good immunotherapeutic drugs, but there is no definite clinical method to predict therapeutic response. The expression of PD-L1 in tumors is considered to be a good indirect reflection of the therapeutic effect (26). We observed a significant positive correlation between PD-L1 protein expression and mRNAsi values (**Figure 3D**). PD-L1, CD11b, F4/80, CD 206, CD 163, FAP, and α-SMA expression levels were divided into high and low groups according to the median values. The correlations between mRNAsi values and immune cell types, which were classified based on the expression of PD-L1 and characteristic markers, were analyzed using the chi-square test. Increases in mRNAsi values were found to be associated with significantly depressed marker expression and increased PD-L1 expression (**Figure 3E**). The mRNAsi seemed to be better at distinguishing CAF subtypes than macrophage subtypes. Overall, our analysis indicates that mRNAsi values positively correlate with PD-L1 expression and negatively correlate with macrophages and CAFs. We know that macrophages, especially M2 macrophages (27), and CAFs (28, 29) play important roles in driving the progression of GC. By evaluating the numbers of these two types of cells, the outcome of GC can be better predicted. The above results suggest that mRNAsi values themselves can serve as a novel predictive biomarker of immunotherapy response.

### Using the mRNAsi to Evaluate the Gene Mutation Status and Reveal a Tumor Signaling Pathway

First, based on the TCGA cohort, the top 10 mutated genes in GC and their mutation rates were obtained (**Figure 3G**). To evaluate whether the mRNAsi can be used as a predictor of the gene mutation status, we analyzed the correlations between somatic mutations in the top 10 mutated genes and the mRNAsi. Strong associations were found between the mRNAsi and the subtypes of mutations in the genes. The mRNAsi values of the mutant subtype group were significantly higher than those of the wild-type subtype group (*P*<0.05), so we could use mRNAsi values to indirectly predict the mutation status of genes (**Figure 3F** and **Supplementary Figure 1B**). Additionally, the sample TMB could be calculated according to the status of gene coding errors, substitutions, deletions, insertions, etc., which was used to describe the mutation density of a gene. Similar to the trend in the single-gene mutation status, the mRNAsi values of the high TMB group were also increased significantly (*P*<0.0001) (**Figure 3H**). The mRNAsi was effective in evaluating prognosis, immune cell infiltration and the gene mutation status. Finally, we used GSEA to explore the possible signaling pathways involved in the progression of GC according to mRNAsi values. In addition to remarkable enrichment of epithelial-mesenchymal transition (EMT), enrichment of the hedgehog signaling pathway, inflammatory response, and Kras signaling was also observed (**Figure 3I**). Previous studies have shown low epithelial subtype genomic integrity and high mesenchymal subtype genomic integrity, and mesenchymal subtypes exhibit low mutational rates and microsatellite stability (30). This study also compared whether the mRNAsi can distinguish key markers of EMT. The results showed that the expression of epithelial subtype markers was positively correlated with mRNAsi values but negatively correlated with mesenchymal subtypes (**Supplementary Figure 1C**). The results of this study were consistent with those of previous studies. In short, combined with the results of our previous analysis, low mRNAsi values were associated with EMT promotion, low mutation rates, microsatellite stability, and a poor prognosis, and these results were consistent with the results of Cheul Oh et al. (30).

### Identification of Differentially Expressed Genes and Construction of Co-Expression Modules

**Figure 4:**
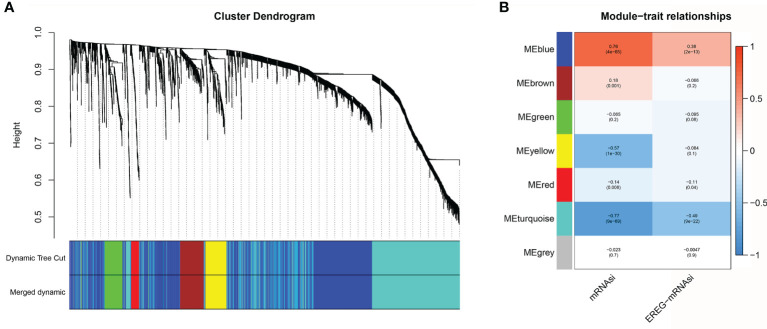
Co-expression module construction. **(A)** Clustering dendrograms of genes. Genes with the same expression pattern were clustered in the same color. **(B)** Module-trait associations. Seven modules were significantly associated with the mRNAsi.

The calculation of mRNAsi values cannot be practically applied in the clinic due to the high number of reference datasets used. Here, we used a variety of algorithms to gradually reduce dimensionality in the hope that key mRNAs would be found to establish a prediction model that was highly similar to the mRNAsi prediction model. First, the “limma” package was used to search for differentially expressed genes between normal tissue and tumor tissue. Under the threshold conditions of |logFC|>1 and adj. Pvalue<0.05, a total of 3099 mRNAs were selected. Based on the 3099 mRNAs and mRNAsi values, co-expression modules were constructed with the WGCNA algorithm to identify mRNAsi-related modules. The most critical parameter of the soft threshold power was set at 4 to assure integral connectivity of co-expression modules. Seven co-expression modules were constructed and displayed in different colors. Clustering dendrograms clustered genes in common gene expression patterns in the same color module (**Figure 4A**). **Figure 4B** shows that the blue and brown modules were positively correlated with the mRNAsi (MEblue: r = 0.76, *P* = 4e−65; MEbrown: r =0.18, *P* = 0.001). The green, yellow, red, turquoise, and gray modules were negatively correlated with the mRNAsi (MEgreen: r = -0.065, *P*=0.2; MEyellow: r =-0.57, *P* = 1e−30; MEred: r =-0.14, *P*= 0.008; ME turquoise: r =-0.77, *P*= 9e−69; MEgray: r =-0.023, *P*= 0.7) (**Figure 4B** and **Supplementary Table 3**). The genes in the blue module were regarded as positive SI-genes (n=14), and those in the turquoise module were negative SI-genes (n=178).

### Verification of SI-Genes by Single-Sample Gene Set Enrichment Analysis

**Figure 5:**
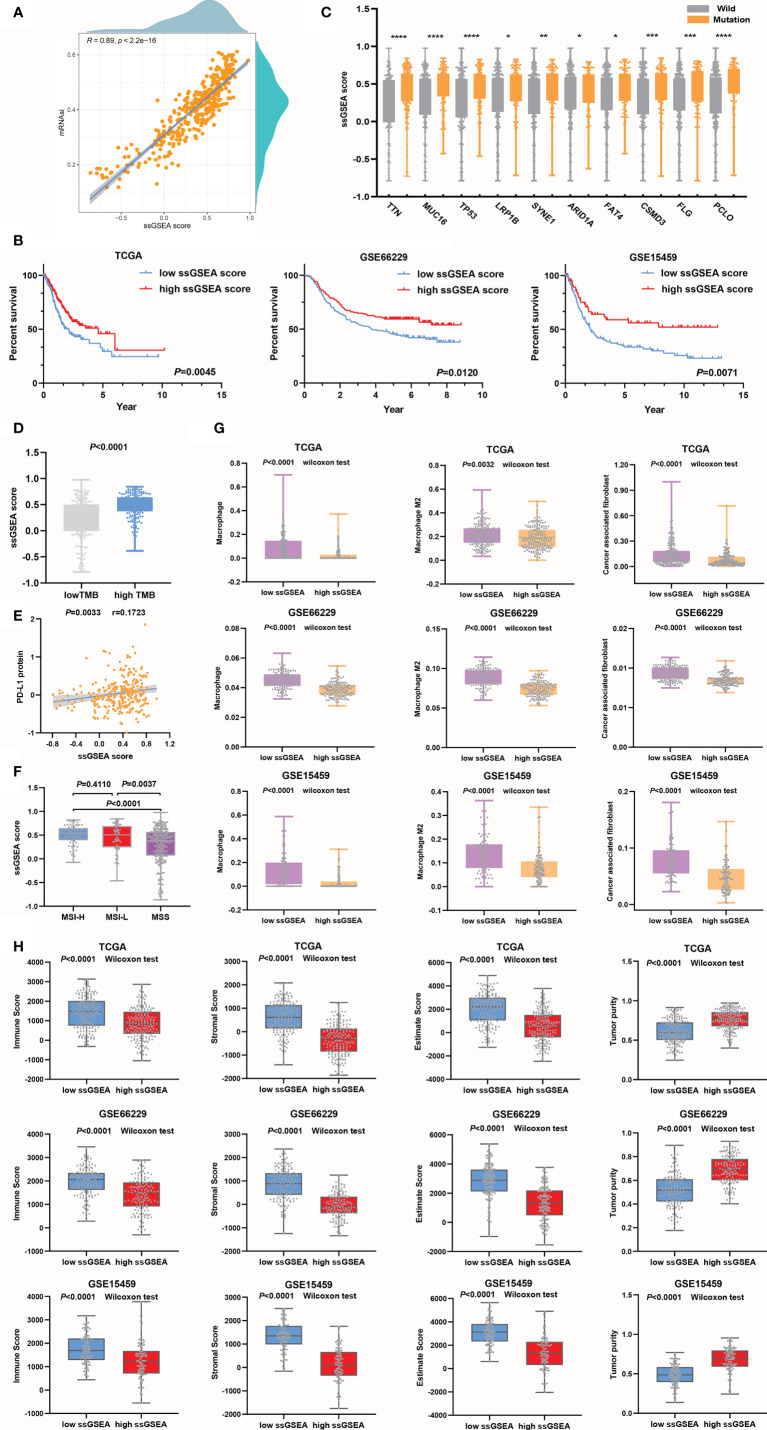
ssGSEA scores were used to evaluate the prognosis and clinicopathological characteristics of GC. **(A)** The association between ssGSEA scores and mRNAsi values. **(B)** The survival time of the GC patients in the high ssGSEA score group was longer than that of those in the low ssGSEA score group in the training and validation cohorts. **(C, D)** Genes in the high ssGSEA score group were more susceptible to mutation than those in the low score group, *P<0.05; **P<0.01; ***P<0.001; ****P<0.0001 **(C)**, and the TMB **(D)** was also higher in the high score group than in the low ssGSEA score group. **(E)** Evaluation of the ssGSEA score as a predictor the efficacy of anti-PD-L1 immunotherapy. **(F)** The ssGSEA score was a good predictor of MSI. **(G)** Assessment of infiltrating macrophage and CAF stratification by ssGSEA scores. **(H)** Assessment of tumor microenvironment components by ssGSEA scores.

Here, ssGSEA was applied to estimate the score of each sample based on positive or negative genes. The SI-gene-based ssGSEA score of each sample was calculated as the positive gene score minus the negative gene score. Pearson’s correlation analysis was performed to verify whether ssGSEA scores were in agreement with mRNAsi values. The results showed a powerful correlation between ssGSEA scores and mRNAsi values (r=0.89, *P*<0.0001) (**Figure 5A**). Then, the value of ssGSEA scores in the prognostic evaluation of GC was verified. We noted that ssGSEA scores could predict the OS of GC accurately in the training cohort (**Figure 5B**). The same results were obtained in the validation cohorts (**Figure 5B**).

The ssGSEA scores of the high TMB group were higher than those of the low TMB group, and the results were congruous with the prediction based on mRNAsi values (**Figure 5D**). The ssGSEA score could distinguish the mutation status very well, especially for the TP53 subtype, producing better results than the mRNAsi (**Figure 5C**). Then, we noticed that the association between ssGSEA scores and PD-L1 protein expression(**Figure 5E**) was higher than that between PD-L1 protein expression and mRNAsi values (**Figure 3B**), which suggested that ssGSEA scores could better evaluate the efficacy of immunotherapy. Next, by comparing the correlations between ssGSEA scores and MSI or MSS in the TCGA cohort, it was found that the ssGSEA scores in the MSS group were significantly lower than those in the MSI-H and MSI-L groups and that ssGSEA scores could clearly predict the MSI status of patients (**Figure 5F**). However, ssGSEA scores did not distinguish between MSI-H and MSI-L. In the process of evaluating EMT, we found that in the training and validation cohorts, the expression of epithelial markers in the high ssGSEA score group was increased, while that of mesenchymal markers was downregulated (**Supplementary Figure 2A**).

We tested the value of ssGSEA scores for predicting infiltrating macrophage and CAF abundances, which were low in the high ssGSEA score group compared to the low group (**Figure 5G**). Surface markers were also detected to support our conclusion (**Supplementary Figure 2B**). The results suggested that the ability of ssGSEA scores to evaluate the immune components of tumors was not lower than that of the mRNAsi. From the perspective of cell infiltration abundances and marker differences, it seemed that ssGSEA scores were more effective in evaluating CAFs than macrophages, and this phenomenon was also seen with the mRNAsi. Finally, high correlations between ssGSEA scores and tumor microenvironment components were observed (**Figure 5H** and **Supplementary Figure 2C**). In conclusion, the ssGSEA score we created based on SI-genes was equivalent to the mRNAsi in predicting the prognosis of GC and immune cell infiltration. Therefore, the screened SI-genes were potential prognostic markers identified through the WGCNA algorithm.

### Construction of a Prognostic Signature

**Figure 6:**
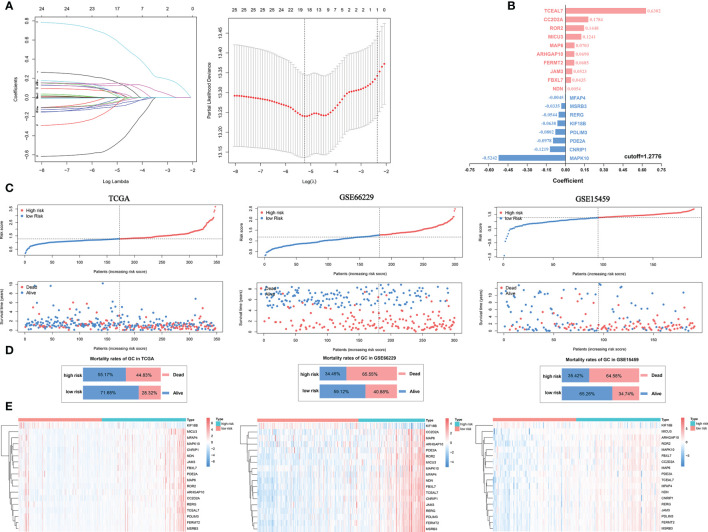
Construction of a prognostic signature for GC. **(A)** Eighteen SI-genes were selected to construct the signature by the logistic Cox regression algorithm. **(B)** The coefficients of the 18 SI-genes. **(C)** The distribution of the riskScore and survival status of patients. **(D)** The overall mortality rates of GC patients in the TCGA, GSE66229, and GSE15459 cohorts. **(E)** The differential expression of the 18 SI-genes in the high- and low-risk groups.

The abilities of positive SI-genes (n=14) and negative SI-genes (n=178) to predict GC outcomes and evaluate immune cell infiltration were indicated by the ssGSEA score. However, many of the included genes were still not convenient for clinical application. Here, we first performed univariate Cox analysis of 192 SI-genes and found that 25 SI-genes were statistically significant in the TCGA cohort (**Supplementary Figure 3A**). Then, the logistic Cox regression algorithm was used to select 18 SI-genes (NDN, ARHGAP10, FERMT2, KIF18B, CC2D2A, RERG, MSRB3, TCEAL7, MAP6, MAPK10, CNRIP1, PDLIM3, ROR2, JAM3, FBXL7, PDE2A, MFAP4, and MICU3) to construct a prognostic risk signature based on the minimum partial likelihood deviance (**Figure 6A**). The coefficient (**Figure 6B**) was multiplied by the expression of each gene, and their sum was considered the riskScore for each sample. According to the median value of the riskScore (cut-off=1.2776), which was used as the cut-off value, the TCGA cohort samples were divided into high- and low-risk groups. Next, the same coefficients and cut-off were applied to the GSE66229 and GSE15459 cohorts. The GSE66229 cohort was divided into 119 high-risk samples and 181 low-risk samples. The GSE15459 cohort was divided into 96 high-risk samples and 95 low-risk samples (nine samples were removed due to lack of follow-up information). We noticed that the proportion of deaths in the samples with a riskScore higher than the cut-off value was increased based on the distribution of the riskScore and survival status (**Figures 6C, D****)**. Finally, we analyzed the expression of the 18 genes included in the signature in the high- and low-risk groups, of which KIF18B was expressed at low levels in the high-risk group, while the other genes were all highly expressed (**Figure 6E**).

### The Prognostic Signature Is Related to Clinical Characteristics and the Immune Response

**Figure 7:**
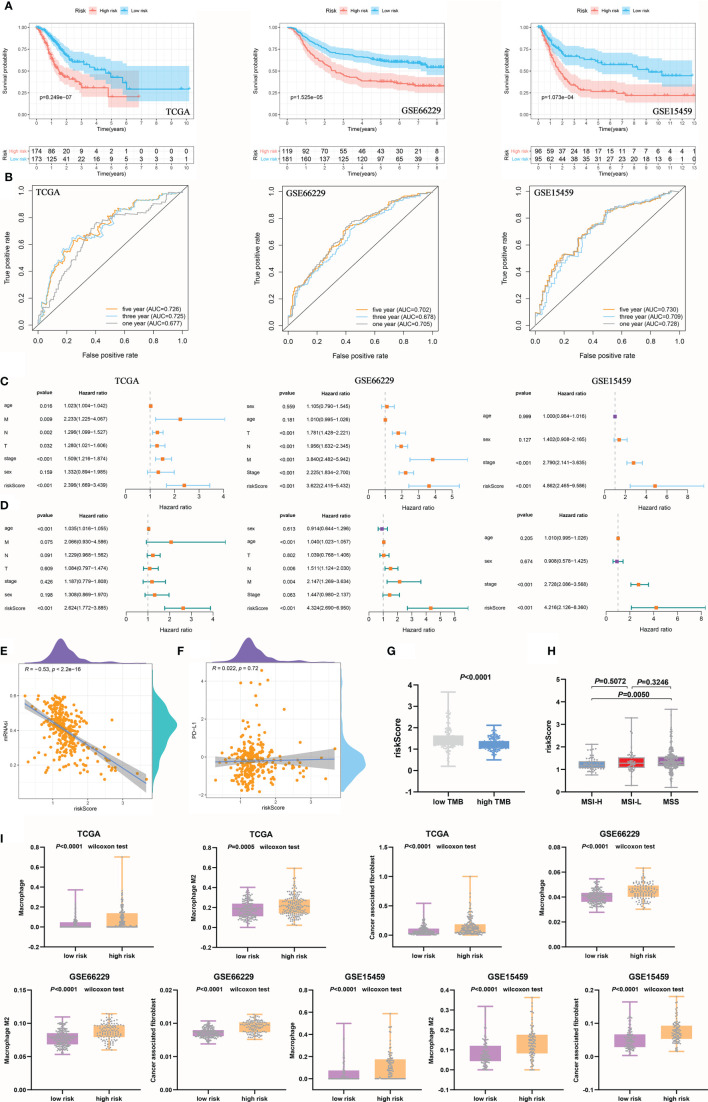
The prognostic signature was related to clinical characteristics and immune cells. **(A)** The survival time of the high-risk group was significantly shorter than that of the low-risk group. **(B)** The ROC curve shows the diagnostic value of the riskScore for GC prognosis. **(C, D)** Univariate **(C)** and multivariate **(D)** Cox regression analyses suggested that the riskScore was an independent prognostic factor. **(E)** The negative correlation between the riskScore and mRNAsi. **(F)** There was no significant correlation between the riskScore and PD-L1 expression. **(G)** The high TMB group corresponded to a lower riskScore than the low TMB group. **(H)** Differences in the riskScore among MSS and MSI groups. **(I)** Assessment of immune cell infiltration abundance by the riskScore.

After the risk groups for the training and validation cohorts were selected based on the cut-off, the value of the riskScore for GC needed to be tested. First, Kaplan-Meier curve analysis was conducted to determine the difference in GC survival between the high- and low-risk groups. The results showed that the survival time of the high-risk group was significantly shorter than that of the low-risk group in the training and validation cohorts (**Figure 7A**). The 3- and 5-year AUCs were 0.725 and 0.726, respectively, in the training cohort. The 1- and 5-year AUCs were 0.702 and 0.702, respectively, in the GSE66229 cohort. In the GSE15459 cohort, the AUCs for 1, 3, and 5 years were 0.728, 0.709, and 0.730, respectively (**Figure 7B**). Univariate Cox regression analysis showed that the riskScore was an important risk factor for GC (HR>1, *P*<0.001) (**Figure 7C**). Additionally, using multivariate Cox analysis, the riskScore was found to be an independent prognostic factor for GC patient survival (HR>1, *P*<0.001) (**Figure 7D**). Both univariate and multivariate Cox regression analyses indicated that the HR value of the riskScore was greater than that of tumor stage, which showed that the riskScore was a better predictor of a poor prognosis in GC than was the tumor stage.

Subsequently, a negative correlation was observed between the riskScore and mRNAsi, but the correlation was much weaker than that between the mRNAsi and ssGSEA score (**Figure 7E**). Accordingly, the riskScore was found to be less valuable in predicting the efficacy of anti-PD-L1 immunotherapy (**Figure 7F**). However, the riskScore was a perfect predictor of the TMB, and the high TMB group had a lower riskScore than the low TMB group (**Figure 7G**). In addition, the riskScore was higher in MSS patients than in MSI-H patients, but there were no statistically significant differences between MSS and MSI-L patients (**Figure 7H**). Pearson’s analysis was performed to analyze the correlations between the riskScore and tumor microenvironment components, and the riskScore was found to be related to stromal components and tumor purity in the training and validation cohorts. However, there were no statistically significant differences in immune components found in the TCGA or GSE15459 cohort (**Supplementary Figure 3B**). Finally, we further explored the infiltration of immune cells in different risk groups. The infiltration abundances of macrophages, M2 macrophages, and CAFs in both the training and validation cohorts were higher in the high-risk group than in the low-risk group (**Figure 7I** and **Supplementary Figure 3C**). In short, the prognostic signature we developed had perfect predictive value for GC prognostic evaluation, immune cell infiltration, the TMB, and microsatellite instability, but its ability to predict PD-L1 blockade response was not as great as that of the ssGSEA score we established earlier.

### Prognostic Value of the Eighteen SI-Gene-Based Signature

**Figure 8:**
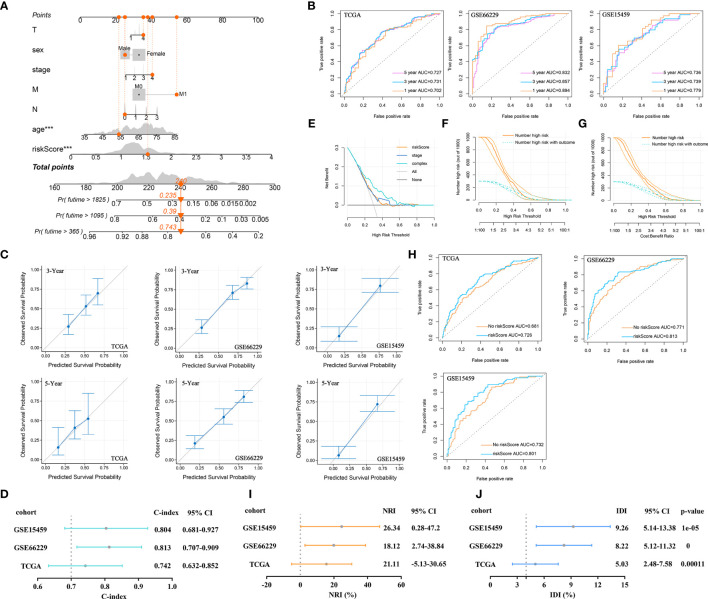
Prognostic value of the established signature. **(A)** The nomogram of clinical characteristics and the riskScore. **(B)** ROC curve analysis for OS prediction by the nomogram in the training and validation cohorts. **(C)** Calibration curve of the nomogram for predicting the OS rates of GC patients in the training and validation cohorts. **(D)** The C-index was plotted to reflect the ratio of the predicted result to the actual result. **(E)** DCA was performed to describe the NB of evaluating the outcome. **(F)** CIC for the clinical characteristics-based risk model. Among 1,000 patients, the dark orange solid line shows the total number of patients considered high risk at each risk threshold. The dark turquoise dashed line shows how many of the high-risk patients would be positive. **(G)** CIC for the risk model based on clinical characteristics and the riskScore. **(H)** ROC curve analysis of OS predictions by the nomogram with or without inclusion of the riskScore. **(I)** The NRI evaluated the predictive power of old and new models. **(J)** IDI evaluated the overall improvement in the model after introducing the riskScore indicator.

At present, the pathological characteristics of patients are commonly used in the clinic to roughly evaluate patient outcomes, but disappointingly, accurate prediction cannot be achieved. Here, the developed risk signature was used in combination with pathological characteristics to predict 1-, 3-, and 5-year OS. First, 1-, 3-, and 5-year OS rates were marked in a nomogram, which was established based on the riskScore and clinicopathological characteristics (**Figure 8A**). According to the nomogram, when the total score was 240, the 1-year OS rate was 74.3%, the 3-year OS rate was 39%, and the 5-year OS rate was 23.5%. The AUCs for the OS predictions for 1, 3, and 5 years were 0.702, 0.731, and 0.727, respectively, for the constructed nomogram in the training cohort. The predictive value of the nomogram was verified in the validation cohorts. In the GSE66229 cohort, the AUCs for the OS predictions for 1, 3, and 5 years were 0.894, 0.857, and 0.832, respectively. In the GSE15459 cohort, the AUCs for OS predictions for 1, 3, and 5 years were 0.779, 0.739, and 0.736, respectively (**Figure 8B**). The calibration curves for this nomogram showed that the predicted survival probabilities at 3 and 5 years were the same as the observed survival probabilities in the training and validation cohorts. Therefore, the established nomogram was relatively reliable in predicting the prognosis of GC (**Figure 8C**). The C-index was used to reflect the ratio of the predicted results to the actual results, which was used to evaluate the predictive ability of the model. The C-indexes of the nomogram for the TCGA, GSE66229, and GSE15459 cohorts were 0.742, 0.813, and 0.804, respectively (**Figure 8D**).

Here, we focused on analyzing the role of the riskScore in the prognostic efficiency of the nomogram by using some evaluation indicators. DCA results were plotted to describe the net benefit (NB) of evaluating the outcome of GC patients using the riskScore, tumor stage, or a combination of all features. The NB of using the riskScore to predict GC outcomes was similar to that of using tumor stage in the training cohort. However, combining the riskScore with tumor stage, T stage, M stage, N stage, age, and sex significantly increased the NB when the threshold was approximately 0.1-0.7 (**Figure 8E**). On this basis, the CIC was drawn to show the proportion of true-positive patients using clinical characteristics and the riskScore. As shown in **Figure 8F**, when the risk threshold was 0.2, approximately 750 patients were classified as high risk by clinical characteristics. Only 280 patients were real outcome cases. However, when we included the riskScore in the model and plotted the CIC for the riskScore combined with clinical characteristics, we found that when the risk threshold was 0.2, approximately 650 patients were classified as high risk by the combined index, and only 250 patients were true outcome cases (**Figure 8G**). The results suggested that the riskScore could improve the prediction of clinical risk stratification for GC.

Next, a ROC curve was plotted to observe the predicted value of the nomogram with or without the riskScore. After the riskScore was added to the predictive model, the AUC showed corresponding improvements in the training and validation cohorts (**Figure 8H**). Sometimes, when a new index is introduced into a prognostic model, the AUC is not significantly improved, and the incremental increase in the AUC is not significant. In this case, the NRI was required to compare the predictive abilities of the old and new models. The NRI showed that after the introduction of the riskScore, the ability of the nomogram to predict OS improved (NRI>0) in the training and validation cohorts (**Figure 8I**). Finally, IDI was used to investigate the overall improvement in the model. After introducing the riskScore, the predictive power of the nomogram was improved by 5.03%, 8.22%, and 9.26%, which were statistically significant increases (*P*<0.001), in the training and validation cohorts (**Figure 8J**).

## Discussion

Based on the role of the mRNAsi across cancers reported by Malta et al. (15), this study further explored the relationships between SI-genes and clinical characteristics, somatic mutations, the tumor microenvironment, immune cell infiltration and a prognostic signature from the perspective of GC by ssGSEA and LASSO Cox regression. Our study found that the ssGSEA score could clearly predict clinical characteristics, somatic mutations, immunotherapeutic responsiveness, tumor microenvironment composition, and macrophage and CAF infiltration in the training and validation cohorts. Finally, to improve application in the clinic, a prognostic signature was constructed based on 18 prognostic SI-genes. ROC curves, calibration curves, DCA, the C-index, CICs, the NRI, and IDI were used to verify that the constructed prognostic model could perfectly predict OS. Thus, our study suggests that the screened SI-genes play important roles in the progression of GC and can be used as important reference markers for further research on tumor cell stemness.

Chemotherapy resistance and early lymph node and peritoneal metastasis are the main causes of a poor prognosis in GC. Studies have suggested that the continuous proliferation and activation of CSCs promote the immortalization of tumor cells (31). The signaling pathways (12, 32) and markers (31, 33) associated with GC CSCs have been widely reported, but no consensus has been reached on the specific mechanism. In addition to Malta et al., Alex et al. (34) inferred cancer stemness using ssGSEA. Zheng et al. (35) provided a stemness index based on relative expression orderings (REOs). The shortcoming was that the previous stemness index was aimed at studying pan-cancer datasets, so no stemness index study specifically targeting GC was developed. For this reason, we intended to identify SI-genes through machine learning to understand the progression of GC from a new perspective.

In this study, we first retrospectively analyzed the established mRNAsi, which has close associations with the prognosis, clinical characteristics, immune cell infiltration, tumor microenvironment, and immunotherapy responsiveness of GC. Then, WGCNA was applied to screen 14 positive SI-genes and 178 negative SI-genes, which were highly correlated with the mRNAsi. A total of 192 SI-genes were used to calculate the ssGSEA score, which was also associated with prognosis, clinical characteristics, immune cell infiltration, the tumor microenvironment, and immunotherapy responsiveness. Therefore, the SI-genes screened by WGCNA were feasible and could be used for further analysis. Then, we unexpectedly found that the ssGSEA score was even better than the mRNAsi in assessing the mutation status of TP53. The mutation rate of the tumor suppressor gene TP53 is 46% in GC. Deletion of TP53 may upregulate vascular endothelial growth factor A (VEGF-A) expression and promote cancer cell angiogenesis, leading to a poor prognosis (36). Gurzu et al. (37) analyzed a large number of human gastric cancer samples and found that mutation of exon 7 in TP53 may induce downregulation of the expression of the tumor suppressor gene Maspin, which leads to GC invasion and metastasis. In addition, TP53 mutations induce hypoxic signaling (38) and inhibit antitumor immunity (39). Through GSEA, we found that the EMT signaling pathway was significantly enriched based on the mRNAsi value. CSCs are an important cause of tumor metastasis and migration, and studies have shown that a mesenchymal phenotype is one of the main features of CSCs (40). Our study reported that mesenchymal marker expression was upregulated in the low ssGSEA score group, while epithelial marker expression was downregulated. The ssGSEA score perfectly evaluated the EMT process. In addition, this study also found that the stemness index was relatively useful for immune response evaluation. PD-L1 protein expression was positively correlated with mRNAsi and ssGSEA scores in the TCGA cohort. In the low mRNAsi and ssGSEA score group, the infiltration of macrophages, M2 macrophages, and CAFs was significantly increased. To make our results more reliable, specific surface markers were also compared, and the results were consistent with the results for cell infiltration. The relationship between CAF infiltration and the stemness index was not reported previously. Here, we found that the ssGSEA score could more accurately assess the abundance of infiltrating CAFs than that of infiltrating macrophages. Macrophages, especially M2 macrophages, are an important cause of tumor cell invasion and EMT (41). Maeda et al. (42) found that stem cell niche factors secreted by CAFs derived from stromal cells conferred tumor invasiveness. Macrophages could induce mesenchymal stem cells to differentiate into a CAF phenotype (43). There are complex connections among macrophages, CAFs, and CSCs. In this study, the ssGSEA score was employed to evaluate the abundances of infiltrating macrophages and CAFs to indirectly predict tumor invasion and metastasis.

Currently, clinicopathological characteristics are commonly used in clinical practice to predict the outcomes of GC patients. However, the predictions are not as accurate as expected (5). Eighteen prognostic SI-genes were used to establish our prognostic signature to improve the accuracy of 1-, 3-, and 5-year OS prediction. Among these 18 genes, Wnt5a-ROR2 signal in GC mesenchymal stem cells, which is associated with enhanced expression of CXCL16 and associated tumor-promoting activity (44). JAM3 is a member of the JAM family. Studies have shown that JAM3 is a new type of surface marker for neural stem cells (45) and an important prognostic marker for haematopoietic stem cells (45). The ZEB1-MSRB3 axis is a vital regulator that maintains the characteristics of breast cancer stem cells and reduces DNA damage during differentiation (46). Next, ROC curves, calibration curves, and the C-index were used to identify a nomogram with strong accuracy for OS prediction. Then, we focused on assessing the value of including the riskScore in the nomogram for OS prediction. DCA, CICs, the NRI, and IDI all showed that the riskScore could significantly improve OS prediction by the nomogram. The established prognostic signature could be of great help for the clinical prediction of GC patient outcomes.

In conclusion, our study described an SI-gene-based ssGSEA score for GC in detail for the first time, which was closely associated with prognosis, clinical characteristics, the TMB, EMT, the immune response, and the tumor microenvironment. The prognostic signature significantly improved OS prediction compared to traditional prediction methods. However, this study was completed at the machine learning level, and further experiments are needed to verify our findings.

## Data Availability Statement

The original contributions presented in the study are included in the article/**Supplementary Material**. Further inquiries can be directed to the corresponding authors.

## Author Contributions

Conceptualization: DM, ZZ, ZW. Data curation: DM, ZZ, SS, DL. Formal analysis: DM, ZZ, SS. Data analysis: DL, SS. Funding acquisition: YH, CZ. Investigation: DM, ZZ, ZW. Methodology: DM, ZZ, SS. Project administration: YH, CZ, ZW. Resources: DM, ZZ. Original draft: DM, ZZ. Writing—review and editing: DM, ZZ. All authors contributed to the article and approved the submitted version.

## Funding

This study was supported by Sanming Project of Medicine in Shenzhen (SZSM201911010), Shenzhen Key Medical Discipline Construction Fund (SZXK016), Shenzhen Sustainable Project (KCXFZ202002011010593), Research start-up fund of part-time PI, SAHSYSU (ZSQYJZPI202001).

## Conflict of Interest

The authors declare that the research was conducted in the absence of any commercial or financial relationships that could be construed as a potential conflict of interest.

## Supplementary Material

Supplementary Table 1 The TMB of each sample in the TCGA cohort.

Supplementary Table 2 The mRNAsi of each sample in the TCGA cohort, Related to **Figure 2**.

Supplementary Table 3 DEGs in different modules, Related to **Figure 4**.

Supplementary Figure 1 Predictive value of mRNAsi, Related to **Figure 3** **(A)** Evaluation of tumor microenvironment components by mRNAsi. **(B)** Evaluation of mRNAsi for gene mutation stratification. **(C)** Differential expression of epithelial and mesenchymal markers in high-low mRNAsi.

Supplementary Figure 2 Predictive value of ssGSEA score, Related to **Figure 5** **(A)** Differential expression of epithelial and mesenchymal markers in high-low ssGSEA score. **(B)** The ssGSEA score was closely related to the expression of surface markers of macrophages and CAFs. **(C)** The ssGSEA score was closely related to tumor microenvironment components.

Supplementary Figure 3 Construction and verification of prognostic signature, Related to **Figures 6** and **7** **(A)** The univariate cox analysis showed that 25 SI-genes were associated with prognosis in the TCGA cohort. **(B)** The riskScore was closely related to tumor microenvironment components. **(C)** The riskScore was closely related to the expression of surface markers of macrophages and CAFs.
