# Supplementary figures and images for: Identification of Stemness Characteristics Associated With the Immune Microenvironment and Prognosis in Gastric Cancer
Source: Front Oncol. 2021 Mar 3;11:626961. doi: 10.3389/fonc.2021.626961 (PMC7966731; doi:10.3389/fonc.2021.626961)

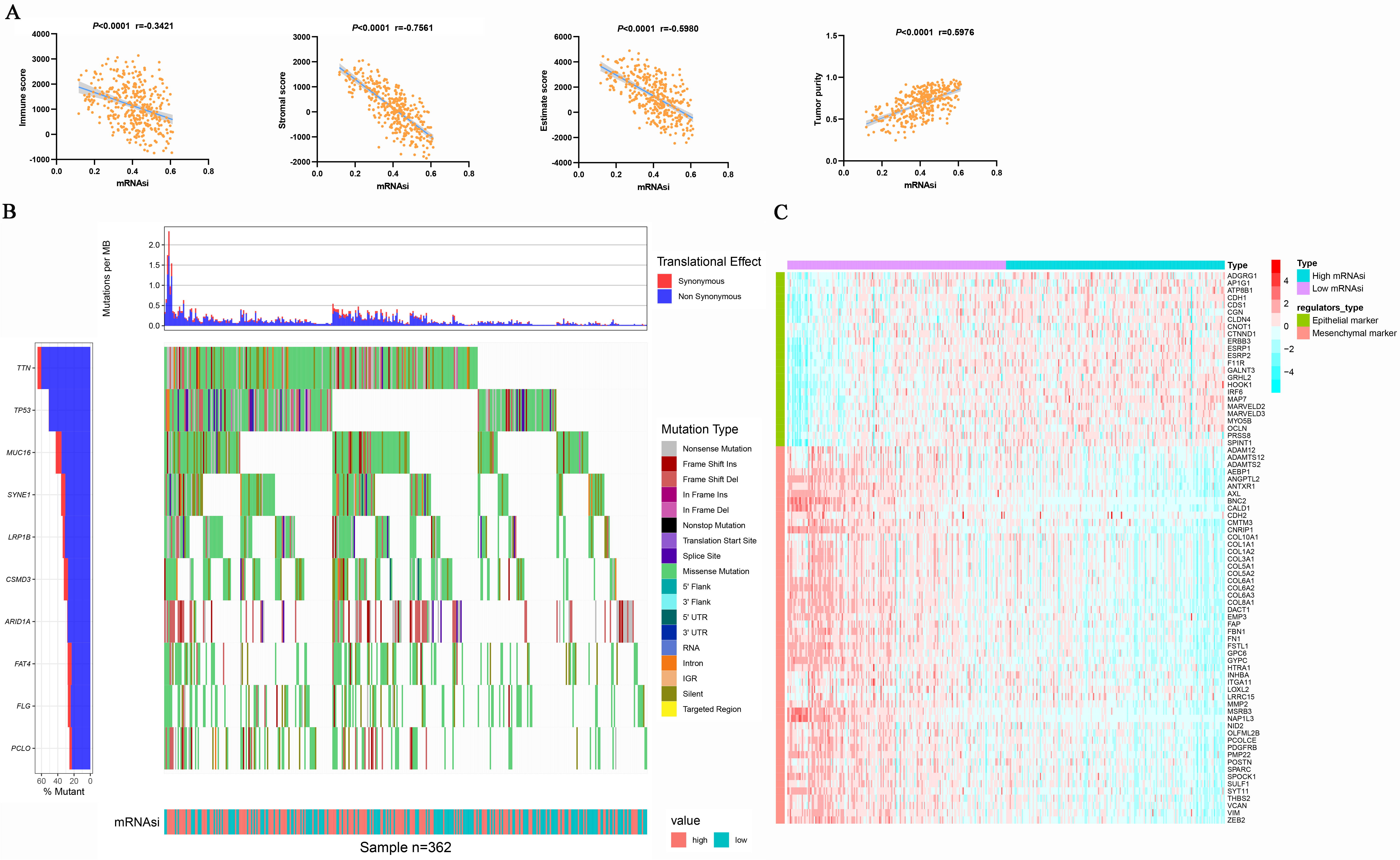

Supplement: Supplementary Figure 1 — Predictive value of mRNAsi, Related to Figure 3 (A) Evaluation of tumor microenvironment components by mRNAsi. (B) Evaluation of mRNAsi for gene mutation stratification. (C) Differential expression of epithelial and mesenchymal markers in high-low mRNAsi. [file Image_1.tif]

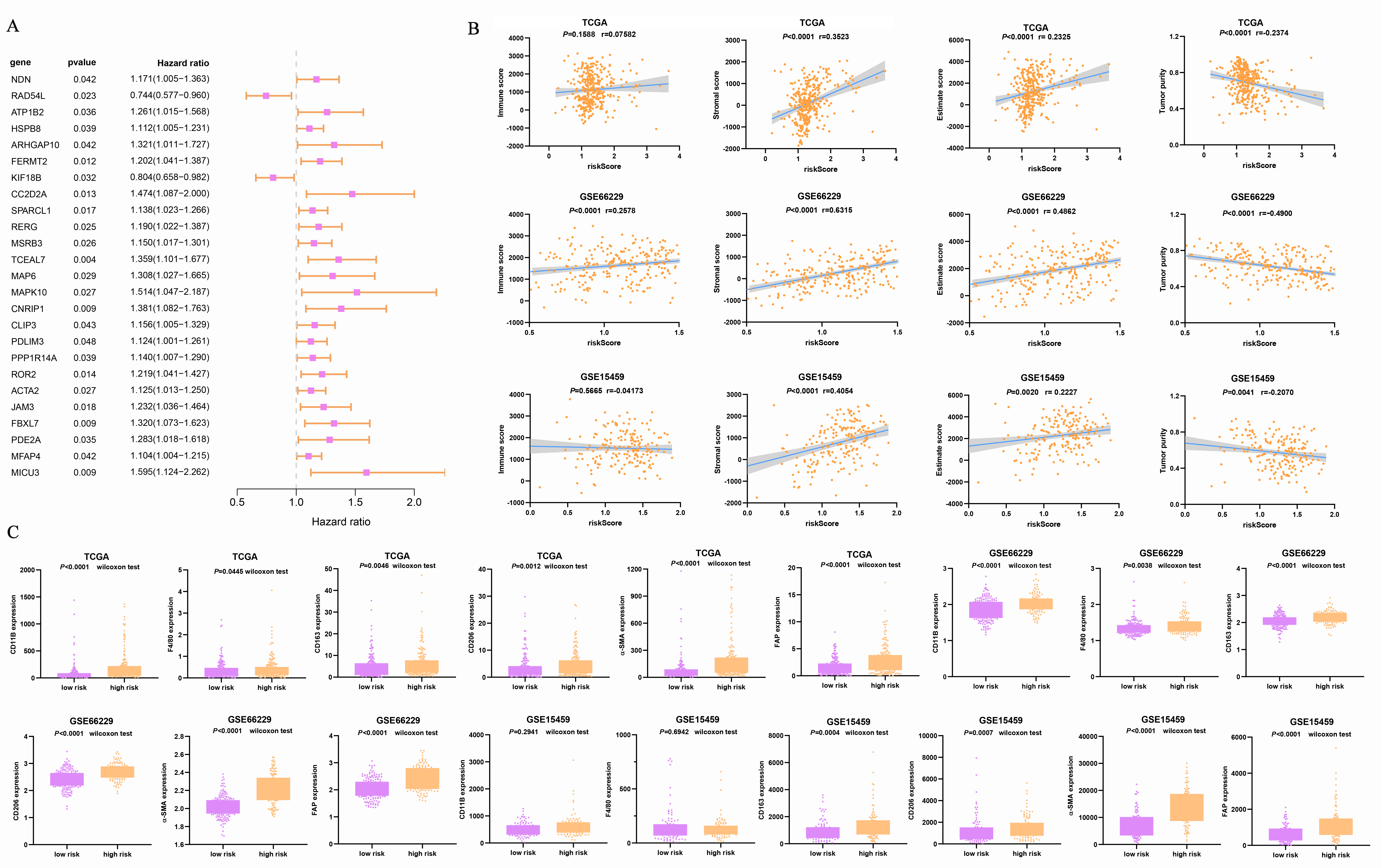

Supplement: Supplementary Figure 3 — Construction and verification of prognostic signature, Related to Figures 6 and 7 (A) The univariate cox analysis showed that 25 SI-genes were associated with prognosis in the TCGA cohort. (B) The riskScore was closely related to tumor microenvironment components. (C) The riskScore was closely related to the expression of surface markers of macrophages and CAFs. [file Image_3.tif]
